# FGF12 induces aberrant mechanosignaling in aortic smooth muscle cells during thoracic aortic aneurysm formation in Marfan syndrome mice

**DOI:** 10.1038/s12276-025-01621-y

**Published:** 2026-01-16

**Authors:** Koung Li Kim, Minju Kim, Yubin Hwang, Duk-Kyung Kim, Jeongmin Kim, June Hyeok Lee, Yong-Wook Son, Jae-Hyung Jang, Kyung-Sun Heo, Misato Iwashita, Yoichi Kosodo, Wonhee Suh

**Affiliations:** 1 College of Pharmacy, Chung-Ang University, Seoul, Republic of Korea; 2 Division of Cardiology, Department of Medicine, Samsung Changwon Hospital, Sungkyunkwan University School of Medicine, Seoul, Republic of Korea; 3 Department of Chemical and Biomolecular Engineering, Yonsei University, Seoul, Republic of Korea; 4 College of Pharmacy, Chungnam National University, Daejeon, Republic of Korea; 5 Neural Regeneration Lab, Korea Brain Research Institute, Daegu, Republic of Korea; 6 R&D Center, GluGene Therapeutics Inc., Seoul, Republic of Korea

**Keywords:** Cell signalling, Aneurysm

## Abstract

Marfan syndrome (MFS), caused by mutations in the *FBN1* gene, predisposes individuals to thoracic aortic aneurysm (TAA), a life-threatening complication. Recent studies have suggested that dysregulated mechanosignaling in aortic smooth muscle cells (SMCs) plays a pivotal role in TAA pathogenesis in MFS. However, the key molecular drivers remain largely undefined. Here we identify fibroblast growth factor 12 (FGF12) as a novel mediator of aberrant mechanosignaling in aortic SMCs during TAA formation in MFS. FGF12 is markedly upregulated in aortic SMCs of thoracic aneurysmal aortas from *Fbn1*^*C1039G/+*^ MFS mice and from patients with MFS. Mechanistically, FGF12 expression is induced by transforming growth factor-β/SMAD signaling and by cyclic mechanical stretch in aortic SMCs. FGF12 upregulates the expression of angiotensin II (AngII) and AngII type 1 receptor (AT1R), thereby activating the AngII/AT1R signaling pathway. FGF12-induced AT1R activation promotes aberrant mechanosignaling, as indicated by increased RhoA-GTP levels, stress fiber formation, focal adhesion assembly and focal adhesion kinase phosphorylation, ultimately leading to increased aortic SMC stiffness. In vivo studies using *Fgf12* heterozygous (*Fgf12*^*+/−*^) mice reveal that *Fgf12* haploinsufficiency significantly ameliorates AngII/β-aminopropionitrile-induced TAA formation, accompanied by reduced AT1R signaling and attenuation of aberrant mechanosignaling in the thoracic aortas. Furthermore, in *Fbn1*^*C1039G/+*^ MFS mice, *Fgf12* haploinsufficiency (*Fgf12*^+/−^*Fbn1*^*C1039G/+*^) substantially mitigates TAA progression and arterial stiffening, while alleviating dysregulated mechanosignaling in thoracic aortic SMCs. Collectively, these findings identify FGF12 as a critical regulator of aberrant mechanosignaling in aortic SMCs and a key contributor to TAA formation in MFS.

## Targeting FGF12 reduces aortic stiffness in Marfan syndrome

Marfan syndrome is a genetic disorder affecting connective tissue, often leading to dangerous aortic problems. This study explores how a protein called FGF12 might contribute to these issues. Researchers found that FGF12 is more active in the aortas of people with Marfan syndrome, which could worsen aortic problems. They used mice to study this further, focusing on how FGF12 affects cells in the aorta. The study involved experiments with mice and human cells to see how reducing FGF12 impacts aortic health. They discovered that lowering FGF12 levels in mice reduced the severity of aortic problems and improved cell function. This suggests that FGF12 plays a significant role in the development of aortic issues in Marfan syndrome. Researchers conclude that targeting FGF12 could be a promising way to treat or prevent these problems in people with Marfan syndrome.

This summary was initially drafted using artificial intelligence, then revised and fact-checked by the author.

## Introduction

Marfan syndrome (MFS) is an autosomal dominant connective tissue disorder that predisposes individuals to thoracic aortic aneurysm (TAA), a life-threatening complication that can progress to aortic dissection and rupture^1^. MFS is caused by mutations in the *FBN1* gene, which encodes fibrillin-1, a major extracellular microfibril component responsible for sequestering latent transforming growth factor-beta (TGF-β) within the extracellular matrix (ECM). Loss or dysfunction of fibrillin-1 disrupts elastin fiber assembly and compromises aortic wall integrity, ultimately leading to progressive aortic dilatation, dissection, or rupture^1,2^. In addition to the impaired structural integrity of the aortic wall, dysregulated TGF-β and angiotensin II (AngII) signaling pathways have been implicated in the pathogenesis of MFS-associated TAA. Excessive TGF-β signaling induces apoptosis and phenotypic modulation of vascular smooth muscle cells (SMCs) and promotes ECM degradation in the aortic wall^3,4^. Moreover, activation of the AngII receptor type 1 (AT1R) signaling pathway has been identified as a pathogenic driver of TAA in MFS, as indicated by the therapeutic efficacy of AT1R blockade in murine MFS models^5^.

Recent studies have highlighted altered mechanosignaling in aortic SMCs of patients with MFS. Aortic SMCs derived from dilated aortas of patients with MFS exhibit increased RhoA-GTP levels, prominent stress fiber formation and enhanced expression of focal adhesion (FA) components compared with those from healthy participants^6^. Similarly, vascular SMCs differentiated from MFS patient-derived induced pluripotent stem cells recapitulate key pathological features of MFS, displaying increased stress fiber formation, FA density and integrin expression^7,8^. These changes collectively contribute to increased cellular stiffness. Cytoskeletal remodeling, particularly stress fiber formation, is a major determinant of vascular SMC stiffness and plays a critical role in generating intracellular mechanical tension. In parallel, FA assembly regulates cell stiffness by promoting cell–ECM adhesion, which limits cellular deformability in response to external mechanical forces. Indeed, aortic SMCs isolated from aneurysmal aortic tissues of patients with MFS are significantly stiffer than those from healthy participants^6^. Importantly, vascular SMCs with increased stiffness exhibit impaired mechanosensation under mechanical stress, contributing to aortic aneurysm formation in AngII-treated mice^9^. Taken together, these findings suggest that aberrant mechanosignaling in aortic SMCs may play a critical role in the pathogenesis of TAA in MFS.

In this study, we identified fibroblast growth factor 12 (FGF12) as a novel regulator of aberrant mechanosignaling and cellular stiffness of aortic SMCs during TAA formation in MFS. *FGF12* belongs to the *FGF* gene family based on sequence and structure similarity^10^. However, FGF12 has distinct biochemical and functional properties compared with other FGFs, as it is neither secreted nor does it activate principal FGF receptors^11^. Our previous work demonstrated that FGF12 upregulates serum response factor (SRF) and its coactivator, myocardin (MYOCD), in human aortic SMCs (HASMCs) in a p38 mitogen-activated protein kinase (p38MAPK)-dependent manner^12^. Given that SRF and its transcriptional cofactors, including MYOCD and myocardin-related transcription factor (MRTF), contribute to vascular SMC stiffening and altered mechanical properties in hypertension and aging, we hypothesized that FGF12 may be involved in the aberrant mechanosignaling during TAA formation in MFS^13,14^. Here, we show that FGF12 expression is upregulated by TGF-β and mechanical stretch and that it promotes aberrant mechanosignaling and increases the stiffness of aortic SMCs through activation of the AngII type 1 receptor (AT1R) signaling pathway. Using *Fgf12* heterozygous (*Fgf12*^*+/−*^) mice, we reveal that *Fgf12* haploinsufficiency significantly ameliorates the AngII/β-aminopropionitrile (BAPN)-induced TAA growth and attenuates aberrant mechanosignaling. Moreover, in a murine model of MFS (*Fbn1*^*C1039G/+*^) carrying the most common class of *Fbn1* mutation causing MFS, *Fgf12* haploinsufficiency mitigates TAA progression and alleviates dysregulated mechanosignaling, highlighting FGF12 as a potential therapeutic target for MFS-associated TAA.

## Materials and methods

### Ethics

All animal procedures were approved by the Institutional Animal Care and Use Committee of Chung-Ang University (approval no. 202301020143) and were conducted in accordance with the guideline for the Care and Use of Laboratory Animals published by the United States National Institutes of Health. Human studies were conducted in accordance with the latest Declaration of Helsinki and were approved by the Institutional Review Board of Samsung Medical Center in Korea (IRB no. 2008-09-026). Written informed consent was obtained from all participants, and all human tissue samples were anonymized before analysis.

### Animals

Experiments were conducted using Sprague-Dawley rats (Orient Bio), C57BL/6 mice (Orient Bio), *Fbn1*^*C1039G/+*^ mice (B6.129-*Fbn1*^*tm1Hcd*^/J; Jackson Laboratory), *Fgf12* heterozygote (*Fgf12*^*+/−*^) mice and sex-matched wild-type (WT) littermates. *Fgf12*^*+/−*^ mice was generated by crossing floxed *Fgf12* mice (*Fgf12*^tm1a(KOMP)Wtsi^; Mutant Mouse Resource & Research Centers)—in which exon 2 of *Fgf12* was flanked by loxP sites—with Sox2-Cre transgenic mice (B6.Cg-*Edil3*^*Tg(Sox2-cre)1Amc*^/J; Jackson Laboratory). *Fgf12*^+/−^ mice were then crossed with *Fbn1*^*C1039G/+*^ mice to establish *Fgf12*^+/−^*Fbn1*^*C1039G/+*^ double-heterozygous mice. All animals were housed in individually ventilated cages under a 12-h/12-h light/dark cycle, with ad libitum access to autoclaved rodent diet and water. Anesthesia was induced via intraperitoneal injection of ketamine (79.5 mg/kg) and xylazine (9.1 mg/kg), with adequacy confirmed by absence of the pedal withdrawal reflex. Euthanasia was performed by cervical dislocation under deep anesthesia.

### Human thoracic aortic tissues

Aneurysmal thoracic aortic tissues were collected at the time of surgical repair from patients diagnosed with MFS according to the revised 2010 Ghent nosology. Non-aneurysmal thoracic aortic control tissue was obtained from an organ transplant donor.

### Reverse transcription polymerase chain reaction (RT–PCR)

Total RNA (1 μg) was reverse transcribed using the Superscript first-strand synthesis kit (Invitrogen). Conventional PCR and real-time PCR were performed using a T100 thermal cycler (Bio-Rad) and a CFX Opus 96 Real-Time PCR System (Bio-Rad) in combination with the Advanced Universal SYBR Green PCR Supermix (Bio-Rad), respectively. Transcript levels were normalized to those of the housekeeping gene *GAPDH*. Primer sequences are listed in Supplementary Table 1.

### Western blotting

Proteins were separated by sodium dodecyl sulfate–polyacrylamide gel electrophoresis and transferred onto nitrocellulose membranes. Membranes were blocked with donkey serum (Sigma-Aldrich) or 5% nonfat milk, then incubated overnight at 4 °C with primary IgGs, followed by incubation with horseradish peroxidase-conjugated secondary IgGs. Protein bands were visualized using chemiluminescent reagents (Amersham Biosciences) and imaged using a western blot imaging system (Vilber). Band intensities were quantified using ImageJ software (NIH). The IgGs used in this study are listed in Supplementary Table 2.

### Aortic wall histopathology

Thoracic aortas were fixed with 4% paraformaldehyde, embedded in paraffin and sectioned at 5 μm. Sections were stained with hematoxylin and eosin (H&E, Sigma-Aldrich), Masson’s trichrome (MT, Sigma-Aldrich) or Verhoeff-van Gieson (VVG, Sigma-Aldrich). Images were acquired using an optical microscope (Olympus). Elastic fiber breaks were counted and normalized to tissue area. Collagen deposition was quantified as the blue-stained area (%) relative to total medial area using ImageJ^15^. Analyses were performed on three representative images from two nonconsecutive sections per aorta by two blinded observers.

### Immunostaining

Aortas were embedded in OCT compound (Leica), snap-frozen on dry ice and sectioned. Tissue sections or cultured cells on slides were fixed, permeabilized and blocked, then incubated with primary IgGs overnight at 4 °C, followed by incubation with secondary IgGs. For chromogenic detection, signals were developed using an ABC peroxidase kit (Vector Laboratories) with 3,3′-diaminobenzidine tetrahydrochloride as substrate. For immunofluorescence, slides were mounted with VECTASHIELD containing 4′,6-diamidino-2-phenylindole (DAPI; Vector Laboratories) for nuclear counterstaining. F-actin was visualized with fluorophore-conjugated phalloidin (Invitrogen). Images were obtained using an optical microscope or a confocal laser scanning microscope (Carl Zeiss). All images are representative of three independent experiments. The IgGs used in this study are listed in Supplementary Table 2.

### Cell culture and treatment

HASMCs (ScienCell Research Laboratories) were cultured in smooth muscle growth medium (SMGM, ScienCell Research Laboratories) and used between passages 3 and 7. Cells were treated as indicated with recombinant human TGF-β (10, 20 ng/ml; R&D Systems), SB431542 (10 μM; Sigma-Aldrich), losartan (10 μM; Sigma-Aldrich), PF573228(10 μM; Sigma-Aldrich) and Y27632 (10 μM; Sigma-Aldrich) in SMGM. Rat and mouse aortic SMCs were isolated from thoracic aortas by collagenase/elastase digestion and cultured in Dulbecco’s modified Eagle medium (Gibco) supplemented with 10% fetal bovine serum (Gibco)^16^. More than 95% of the isolated cells were α-smooth muscle actin positive, and cells were used within three passages.

### Luciferase reporter assay

A 2204-bp fragment of the human *FGF12* 5ʹ-regulatory region (NM_004113.5) was amplified from HASMC genomic DNA and cloned into the pGL3-basic firefly luciferase reporter vector (Promega), generating pGL3-phFGF12. A mutant construct (pGL3-phmFGF12) was created in which the SMAD binding element (CAGACA; positions −411/−406 relative to the transcription start site) was mutated, as described previously^17^. HASMCs were cotransfected with either empty plasmid (pGL3-basic), pGL3-phFGF12 or pGL3-phmFGF12, along with a *Renilla* luciferase control plasmid (pRL-SV40; Promega), using Lipofectamine 2000 (Invitrogen). After 48 h, luciferase activity was measured using a dual-luciferase assay kit (Invitrogen). Firefly luciferase activity was normalized to *Renilla* luciferase activity in each sample.

### Cyclic mechanical stretch

Rat aortic SMCs were plated on type IV collagen-coated BioFlex 6-well flexible-bottom culture plates (Flexcell International) and subjected to cyclic stretch (10% or 20% elongation, 1.0 Hz) using an FX-4000T FlexCell Tension Plus system (Flexcell International). Cells were stretched for the indicated durations in medium containing 20% fetal bovine serum.

### Adenovirus production and transduction

Human *FGF12* cDNA has 100% amino acid homology with its murine homologs. Human *FGF12* cDNA (RC215868; OriGene) was used to construct a recombinant adenoviral vector. Recombinant adenovirus expressing FGF12 (Ad *FGF12*) and a control adenovirus expressing β-galactosidase (Ad *LacZ*) were produced and purified by a commercial service (ViraQuest) as described previously^17^. HASMCs were transduced with Ad *FGF12* or Ad *LacZ* in smooth muscle basal medium (SMBM, ScienCell Research Laboratories) for 6 h. After replacing with fresh SMGM, cells were cultured for an additional 72 h before analysis.

### ELISA for AngII

AngII concentrations in culture media were measured using a human AngII ELISA kit (Antibodies.com). Conditioned media from HASMC cultures were collected and processed according to the kit instructions. After binding, washing and sequential incubation with enzyme-conjugated detection IgGs and substrate, absorbance was measured at 450 nm using a microplate reader (Promega).

### Adeno-associated virus (AAV) sh*FGF12* production and transduction

A short-hairpin RNA (shRNA) targeting FGF12 (sh*FGF12*) was designed on the basis of the NCBI RefSeq for *FGF12* (Gene ID: 2257). The target sequence (5′-AGAACCATCGCTACATGAAAT-3′) was selected using an shRNA design tool (VectorBuilder) and corresponds to a validated sequence from the RNAi Consortium (TRCN0000425873). A hairpin construct was synthesized as 5′-AGAACCATCGCTACATGAAAT-CTCGAG-ATTTCATGTAGCGATGGTTCT-TTTTTT-3′. The sh*FGF12* cassette (U6 promoter-sh*FGF12*-poly(T)) was synthesized by a commercial service (Macrogen) and inserted into the pAAV-MCS vector (VPK-410; Cell Biolabs). AAV particles carrying sh*FGF12* or a control shRNA were produced in HEK293 cells using a helper-free triple plasmid transfection method as described previously^18,19^. In brief, a pHelper plasmid (Stratagene), AAV capsid plasmid (pXR1) and the transgene plasmid were cotransfected. At 68–72 h post-transfection, cells were collected and lysed by repeated freeze–thaw cycles, and viral particles were purified by iodixanol (OptiPrep, 1893; Serumwerk) gradient ultracentrifugation. The 40% iodixanol fraction containing AAV was collected and buffer-exchanged using centrifugal filter units (100 kDa cutoff; Millipore). HASMCs were transduced with AAV sh*FGF12* or AAV shControl in SMBM for 6 h. After medium change to fresh SMGM, cells were cultured for an additional 72 h before analysis.

### Active RhoA pulldown assay

Active RhoA (GTP-bound RhoA) was measured using a RhoA activation assay kit (Cytoskeleton). Cell lysates were incubated with Rhotekin RhoA-binding domain agarose beads for 1 h at 4 °C. Beads were washed and then boiled in Laemmli buffer to elute bound proteins. GTP-bound (active) RhoA and total RhoA in the lysates were detected by western blotting.

### Measurement of cellular stiffness using atomic force microscopy (AFM)

Cell stiffness was measured using contact mode AFM (Bioscope Resolve with NanoScope 9.4 software, Bruker) mounted on an inverted microscope (ECLIPSE Ti2, Nikon), following a protocol modified from a previous publication^20^. A silicon nitride cantilever (MLCT-Bio, Bruker) with a pyramidal tip (nominal spring constant 0.02 N/m) was used to indent cells and measure the Young’s modulus. Randomly selected cells were indented at multiple points between the nucleus and cell periphery. Young’s modulus was calculated using the Hertz model from five to ten force curves per indentation point.

### Blood pressure (BP) measurement

BP was measured in conscious mice by tail plethysmography using a BP-2000 system (Visitech Systems)^21–24^. To obtain reliable measurements, mice were acclimated to the apparatus for seven consecutive days before data collection. All BP measurements were performed in the morning, and values were averaged over multiple consecutive readings for each mouse.

### Cardiac function assessment

Mice were lightly anesthetized with 2% isoflurane and placed supine on a temperature-controlled platform. Electrocardiogram electrodes were used to monitor heart rate. Transthoracic echocardigraphy was performed using a VINNO 6 Doppler ultrasound system (VINNO Corporation) with a high-frequency transducer. Left ventricular dimensions were measured from M-mode images obtained in a short-axis view at the mid-ventricular level. Left ventricular internal diameter in diastole was measured, and fractional shortening and left ventricular ejection fraction were calculated. Cardiac output was derived from stroke volume and heart rate, where stroke volume was calculated from the aortic velocity–time integral and the aortic root diameter. All measurements were averaged over three cardiac cycles.

### AngII/BAPN-induced mouse TAA model

TAA was induced by challenging mice with AngII infusion in combination with BAPN administration, as described previously^25^. Twelve-week-old male mice were implanted with osmotic mini-pumps (Alzet, Durect Corp.) delivering AngII (1 μg/kg/min, Sigma-Aldrich) for 4 weeks, and were simultaneously given BAPN (1 g/l; Sigma-Aldrich) in their drinking water for the first 2 weeks. Mice that died before study completion underwent necropsy to confirm aortic rupture as the cause of death.

### Doppler ultrasound imaging and in vivo arterial stiffness measurement

Mice were lightly anesthetized with 2% isoflurane and placed supine on a temperature-controlled platform. Thoracic aortic images were acquired using the VINNO 6 Doppler ultrasound system, which is equipped with an automatic arterial stiffness analysis tool (VINNO Corporation). High-resolution B-mode images were used to measure the maximal diameter of the ascending aorta and to assess TAA incidence. Pulse wave velocity (PWV) was calculated as the distance between carotid and femoral sampling sites divided by the transit time of the pulse wave between those sites.

### Statistical analysis

All data are presented as the mean ± s.e.m. Statistical significance was evaluated using an unpaired Student’s *t*-test or one-way analysis of variance (ANOVA) with Bonferroni’s post-hoc test, as appropriate. TAA incidence was analyzed by Fisher’s exact test. A *P* value of <0.05 was considered significant. Sample sizes (*n*) are indicated in figure legends.

## Results

### FGF12 expression in aortic SMCs of thoracic aneurysmal aortas from *Fbn1*^*C1039G/+*^ mice and patients with MFS

**Fig. 1:**
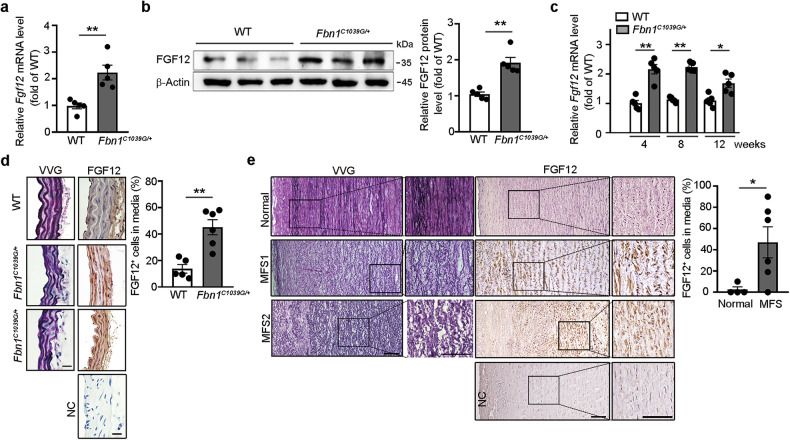
*FGF12* is highly expressed in medial SMCs of thoracic aneurysmal aortas from *Fbn1*^*C1039G/+*^ mice and patients with MFS. **a**,**b** *Fgf12* mRNA and FGF12 protein levels in thoracic aortas from 16-week-old WT and *Fbn1*^*C1039G/+*^ MFS mice. Expression levels are quantified by real-time RT–PCR (**a**) and western blotting (**b**) and data are expressed relative to WT values (set as 1; *n* = 5 mice per group). **c** *Fgf12* mRNA levels in thoracic aortas from WT and *Fbn1*^*C1039G/+*^ mice at 4, 8 and 12 weeks of age. Expression is shown relative to WT at 4 weeks (set as 1; *n* = 5 mice per group). **d** Representative VVG (elastin, black)-stained and immunohistochemical (FGF12, brown) images of thoracic aortas from WT and *Fbn1*^*C1039G/+*^ mice. Quantification of FGF12^+^ cells per total medial cells is shown (*n* = 5 mice per group). Scale bars, 20 μm. **e** Representative VVG-stained and immunohistochemical (FGF12, brown) images of thoracic aortas from a healthy participant and two patients with MFS (MFS1 and MFS2). Quantification of FGF12^+^ cells per total medial cells is shown (*n* = 4–6 samples per group). Scale bars, 100 μm. In **d** and **e** negative control (NC) sections were incubated with nonrelevant primary IgG. All data are presented as mean ± s.e.m. and were analyzed by unpaired Student’s *t*-test (*P < 0.05, **P < 0.01).

We first evaluated FGF12 expression in the thoracic aortas of 16-week-old WT and *Fbn1*^*C1039G/+*^ mice, a widely used murine MFS model carrying the most prevalent *Fbn1* mutation. Quantitative RT–PCR and western blot analyses revealed a significant upregulation of *Fgf12* mRNA and FGF12 protein in *Fbn1*^*C1039G/+*^ mice compared with sex-matched WT littermates (Fig. 1a,b). Notably, *Fgf12* mRNA levels were already significantly elevated in the thoracic aortas of 4-week-old *Fbn1*^*C1039G/+*^ mice compared with WT (Fig. 1c). Histological examination with VVG staining showed severe fragmentation of elastic fibers in the aortic media of *Fbn1*^*C1039G/+*^ mice, whereas WT aortas exhibited intact, well-organized elastic lamellae (Fig. 1d). Immunohistochemical staining further demonstrated prominent FGF12 expression in medial SMCs of thoracic aneurysmal aortas from *Fbn1*^*C1039G/+*^ mice, in contrast to minimal FGF12 expression in WT controls (Fig. 1d). Similarly, in human tissues, immunohistochemistry revealed substantially increased FGF12 expression in medial SMCs of thoracic aneurysmal aortas from patients with MFS compared with a non-aneurysmal control aorta (Fig. 1e and Supplementary Fig. 1).

### *FGF12* is upregulated by TGF-β signaling and by cyclic mechanical stretch in aortic SMCs

**Fig. 2:**
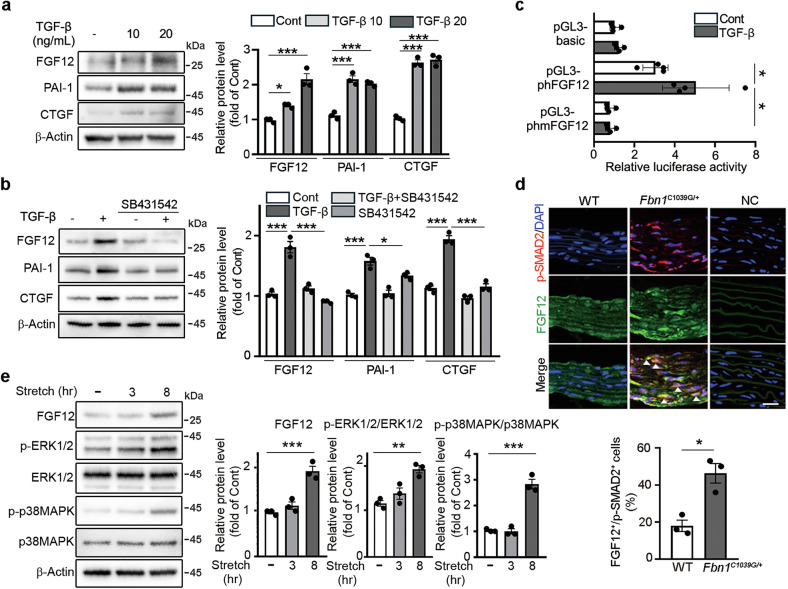
*FGF12* is upregulated by TGF-β/SMAD signaling and mechanical stress in aortic SMCs. **a** TGF-β treatment increases FGF12 expression in HASMCs. **b** Pharmacological inhibition of TGF-β/SMAD signaling with an ALK5 inhibitor (SB431542, 10 μM) attenuates TGF-β-induced FGF12 upregulation. In **a** and **b**, HASMCs were treated with TGF-β for 48 h. Western blots show protein levels of FGF12 and the TGF-β target genes, including PAI-1 and CTGF. Protein levels are quantified relative to untreated controls (Cont, set as 1; *n* = 3 independent experiments). **c** TGF-β enhances *FGF12* promoter activity. HASMCs were transfected with a firefly luciferase vector under control of the human *FGF12* promoter (pGL3-phFGF12), a mutant *FGF12* promoter lacking the SMAD-binding element (pGL3-phmFGF12), or an empty control vector (pGL3-basic), along with a *Renilla* luciferase plasmid for normalization. Cells were treated with or without TGF-β. Firefly luciferase activity is expressed relative to the pGL3-basic control (set as 1; *n* = 4). **d** Representative immunofluorescence images and quantification of phosphorylated SMAD2 (p-SMAD2, red) and FGF12 (green) in the medial layer of thoracic aortas from WT and *Fbn1*^*C1039G/+*^ mice. Colocalized cells (FGF12^+^/p-SMAD2^+^) are indicated by white arrowheads. Nuclei are stained with DAPI (blue). NC was incubated with nonrelevant primary IgG. Scale bars, 20 μm. **e** Cyclic mechanical stretch induces FGF12 expression in rat aortic SMCs. Cells were subjected to 20% cyclic stretch at 1.0 Hz for 0, 3 or 8 h using the FlexCell system. Western blots show protein levels of FGF12, phosphorylated ERK1/2 (p-ERK1/2), total ERK, phosphorylated p38MAPK (p-p38MAPK) and total p38MPAK. Phospho-protein levels were normalized to total protein. Protein levels are quantified relative to unstretched controls (Cont, set as 1; *n* = 3 independent experiments). All data are presented as mean ± s.e.m. and were analyzed by unpaired Student’s *t*-test or one-way ANOVA with Bonferroni’s post-hoc test (*P < 0.05, **P < 0.01, ***P < 0.001).

Building on our previous findings that the *FGF12* promoter contains a SMAD-binding element (CAGACA) located at −411 to −406 upstream of the transcription start site, we hypothesized that TGF-β signaling, which is activated in MFS aortic walls, might regulate FGF12 expression^17^. In HASMCs, TGF-β significantly increased the expression of FGF12, as well as known TGF-β target genes such as plasminogen activator inhibitor-1 (PAI-1) and connective tissue growth factor (CTGF), as shown by RT–PCR and western blot analyses (Fig. 2a and Supplementary Fig. 2a). Pharmacological inhibition of TGF-β/SMAD2/3 signaling with the ALK5 inhibitor (SB431542) substantially abrogated TGF-β-induced upregulation of FGF12 and the TGF-β target genes (Fig. 2b and Supplementary Fig. 2bb). In luciferase reporter assays, TGF-β enhanced the transcriptional activity of the *FGF12* promoter, but it failed to increase the promoter activity of a plasmid with mutations in the SMAD-binding element (Fig. 2c). Immunofluorescence staining of thoracic aortic tissue from *Fbn1*^*C1039G/+*^ mice demonstrated increased FGF12 expression in aortic SMCs colocalized with phosphorylated SMAD2, compared with WT controls (Fig. 2d). Given that cyclic mechanical stretch is markedly increased in the aneurysmal thoracic aortic walls in MFS, we next investigated whether mechanical stress upregulates FGF12. Rat aortic SMCs subjected to pathological levels of cyclic stretch (20% elongation) exhibited a significant induction of FGF12 protein by 8 h of stimulation (Fig. 2e). This was accompanied by enhanced phosphorylation of p38MAPK and extracellular signal-regulated kinase 1/2 (ERK1/2), two key mechanosensitive MAPKs activated by mechanical stress in vascular SMCs^26–28^. By contrast, rat aortic SMCs subjected to physiological levels of cyclic stretch (10% elongation) showed no change in FGF12 expression (Supplementary Fig. 3). These data indicate that FGF12 expression is induced by both TGF-β signaling and pathological cyclic mechanical stretch, two important contributors to TAA pathogenesis in MFS.

### *FGF12* enhances AngII/AT1R signaling in HASMCs

**Fig. 3:**
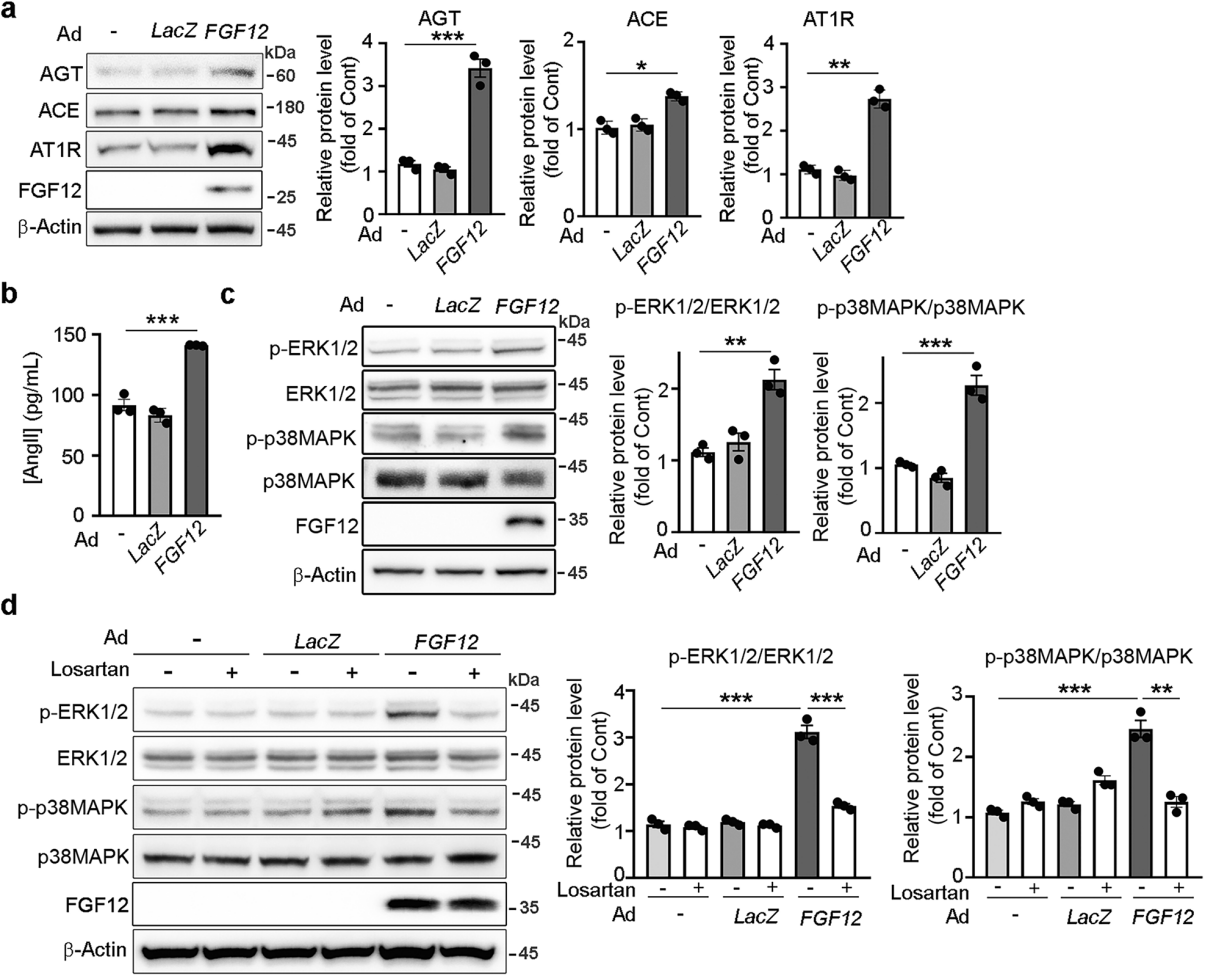
*FGF12* enhances AngII/AT1R signaling in HASMCs. **a** *FGF12* upregulated AGT, ACE and AT1R expression in HASMCs. Western blotting was performed on lysates from untransduced cells and cells transduced with adenovirus expressing FGF12 (Ad *FGF12*) or β-galactosidase (Ad *LacZ*). Protein levels are quantified relative to untransduced controls (Cont, set as 1; *n* = 3 independent experiments). **b** *FGF12* increases AngII production. AngII concentrations in conditioned media from untransduced, Ad *LacZ*-transduced and Ad *FGF12*-transduced cells were measured by ELISA (*n* = 3). **c** *FGF12* enhanced ERK1/2 and p38MAPK phosphorylation. **d** Losartan, an AT1R inhibitor, attenuates *FGF12*-induced ERK1/2 and p38MAPK phosphorylation. In **c** and **d**, western blotting was performed on untransduced and Ad-transduced cells treated with or without losartan. Phospho-protein levels were normalized to total protein. Protein levels are quantified relative to untransduced controls (Cont, set as 1; *n* = 3 independent experiments). All data are presented as mean ± s.e.m. and were analyzed by one-way ANOVA with Bonferroni’s post-hoc test (*P < 0.05, **P < 0.01, ***P < 0.001).

Reciprocal crosstalk between the TGF-β and AngII signaling pathways has been implicated in TAA pathogenesis in MFS. Moreover, mechanical stretch can directly activate AT1R in various cell types, including vascular SMCs^29^. Given that FGF12 expression is upregulated by both TGF-β signaling and mechanical stretch, we investigated whether FGF12 modulates the AngII/AT1R signaling pathway in HASMCs. Adenoviral overexpression of FGF12 (Ad *FGF12*) significantly increased the protein levels of angiotensinogen (AGT), angiotensin-converting enzyme (ACE) and AT1R compared with both untransduced and Ad *LacZ*-transduced cells (Fig. 3a). Consistent with these findings, ELISA demonstreated elevated AngII levels in the culture media of Ad *FGF12*-transduced HASMCs (Fig. 3b). Furthermore, western blot analysis showed enhanced phosphorylation of AT1R downstream signaling molecules, including ERK1/2 and p38MAPK, in Ad *FGF12*-transduced cells (Fig. 3c). shRNA-mediated knockdown of *FGF12* reversed FGF12 overexpression-induced changes in AT1R, AGT and ACE expression, as well as ERK1/2 and p38MPAK phosphorylation, confirming the specific contribution of FGF12 to AngII/AT1R pathway activation (Supplementary Fig. 4). Notably, treatment with losartan, a selective AT1R inhibitor, markedly attenuated FGF12-induced ERK1/2 and p38MAPK phosphorylation (Fig. 3d). These results suggest that FGF12 robustly enhances AngII/AT1R signaling in HASMCs.

### *FGF12* induces aberrant mechanosignaling and increases HASMC stiffness via the AT1R/RhoA/ROCK pathway

**Fig. 4:**
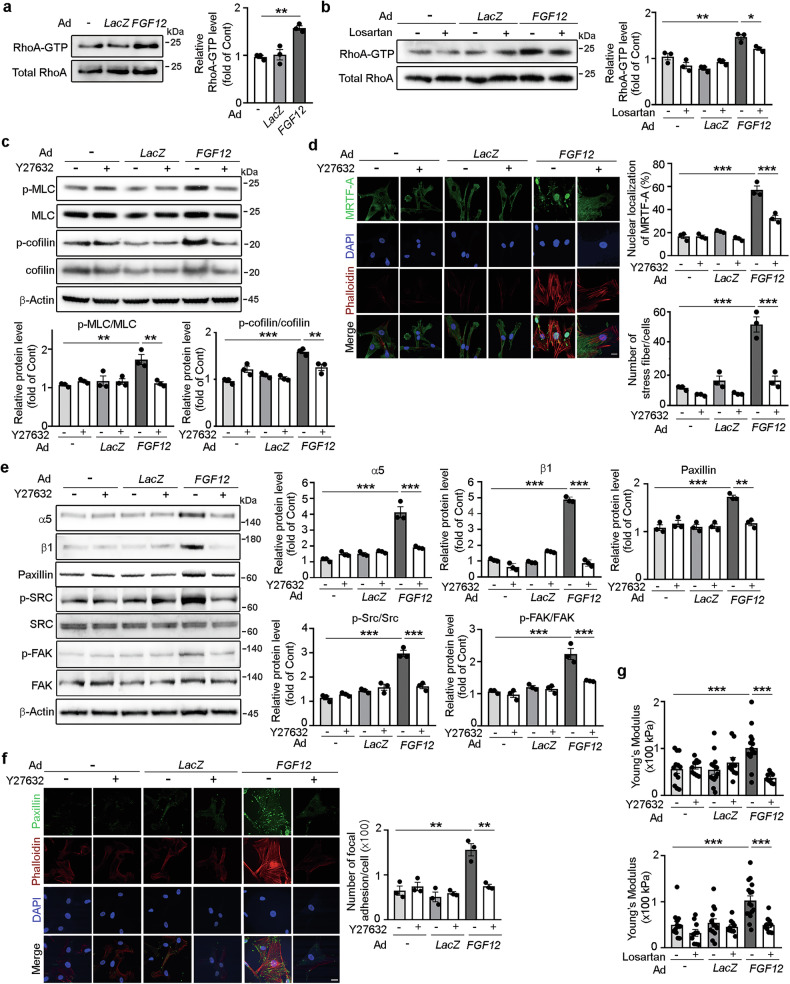
*FGF12* induces aberrant mechanosignaling and increases HASMC stiffness via the AT1R/RhoA/ROCK pathway. **a** *FGF12* induces RhoA activation in HASMCs. Levels of GTP-bound RhoA (RhoA-GTP) were measured by RhoA pulldown assays in untransduced and Ad-transduced HASMCs. **b** Losartan reduces *FGF12*-induced RhoA activation. Following Ad transduction, HASMCs were treated with or without losartan. **c** *FGF12* enhances phosphorylation of MLC (p-MLC) and cofilin (p-cofilin) via ROCK. Western blotting was performed on untransduced and Ad-transduced HASMCs treated with or without the ROCK inhibitor (Y27632). **d** *FGF12* promotes nuclear translocation of MRTF-A and stress fiber formation via ROCK. Following Ad transduction, HASMCs were treated with or without Y27632 and stained for MRTF-A (green) and with phalloidin (red). **e** *FGF12* upregulates integrin α5, integrin β1 and paxillin expression and enhances phosphorylation of SRC (p-SRC) and FAK (p-FAK) via ROCK. Western blotting was performed on untransduced and Ad-transduced HASMCs treated with or without Y27632. **f** *FGF12* promotes FA formation via ROCK. Following Ad transduction, HASMCs were treated with or without Y27632 and stained for paxillin (green) and with phalloidin (red). **g** *FGF12* increases cellular stiffness, which is suppressed by losartan or Y27632. After Ad transduction, HASMCs were treated with or without losartan and/or Y27632. The stiffness of individual cells was measured by AFM nanoindentation and is expressed as Young’s modulus (*n* = 10–15 cells per group). In **a**–**c** and **e**, RhoA-GTP and phospho-protein levels were normalized to total protein. Protein levels are quantified relative to untransduced controls (Cont, set as 1; *n* = 3 independent experiments). In **d** and **e**, images and quantified data are representative of three independent experiments. Nuclei are stained with DAPI (blue). Scale bars, 20 μm. All data are presented as mean ± s.e.m. and were analyzed by one-way ANOVA with Bonferroni’s post-hoc test (*P < 0.05, **P < 0.01, ***P < 0.001).

AngII/AT1R signaling is known to enhance RhoA/Rho-associated kinase (ROCK) activity, a central mediator of mechanosignaling, in vascular SMCs. We therefore examined whether FGF12 regulates the RhoA/ROCK-mediated mechanosignaling pathway in aortic SMCs^30^. Adenoviral FGF12 overexpression significantly increased the levels of GTP-bound active RhoA (RhoA-GTP) in HASMCs, an effect that was abrogated by losartan treatment (Fig. 4a,b). FGF12 overexpression also enhanced the phosphorylation of downstream effectors myosin light chain (MLC) and cofilin, accompanied by phalloidin^+^ stress fiber formation and increased nuclear localization of MRTF-A (Fig. 4c,d; see Supplementary Fig. 5 for enlarged images). These changes were substantially suppressed by Y27631, a selective ROCK inhibitor (Fig. 4c,d). Western blot analysis further revealed that Ad *FGF12*-transduced HASMCs exhibited upregulated expression of FA components, including integrins α5, integrin β1 and paxillin, as well as increased phosphorylation of SRC and FA kinase (FAK), compared with untransduced and Ad *LacZ*-transduced controls (Fig. 4e). These effects were also attenuated by Y27631. Consistently, immunofluorescence staining demonstrated an increased number of paxillin^+^ FAs in Ad *FGF12*-transduced cells, which was reversed by Y27632 (Fig. 4f; see Supplementary Fig. 6 for enlarged images). Given the established roles of stress fibers, FAs and FAK/SRC signaling in regulating cellular stiffness, we assessed cellular stiffness using AFM nanoindentation. FGF12 overexpression significantly increased the Young’s modulus of HASMCs, indicative of elevated cellular stiffness (Fig. 4g). This stiffening effect was significantly attenuated by both Y27632 and losartan. Together, these results demonstrate that *FGF12* induces aberrant mechanosignaling and increases HASMC stiffness via activation of the AT1R/RhoA/ROCK pathway.

### *Fgf12* haploinsufficiency ameliorates TAA formation and attenuates aberrant mechanosignaling in AngII/BAPN-challenged mice

**Fig. 5:**
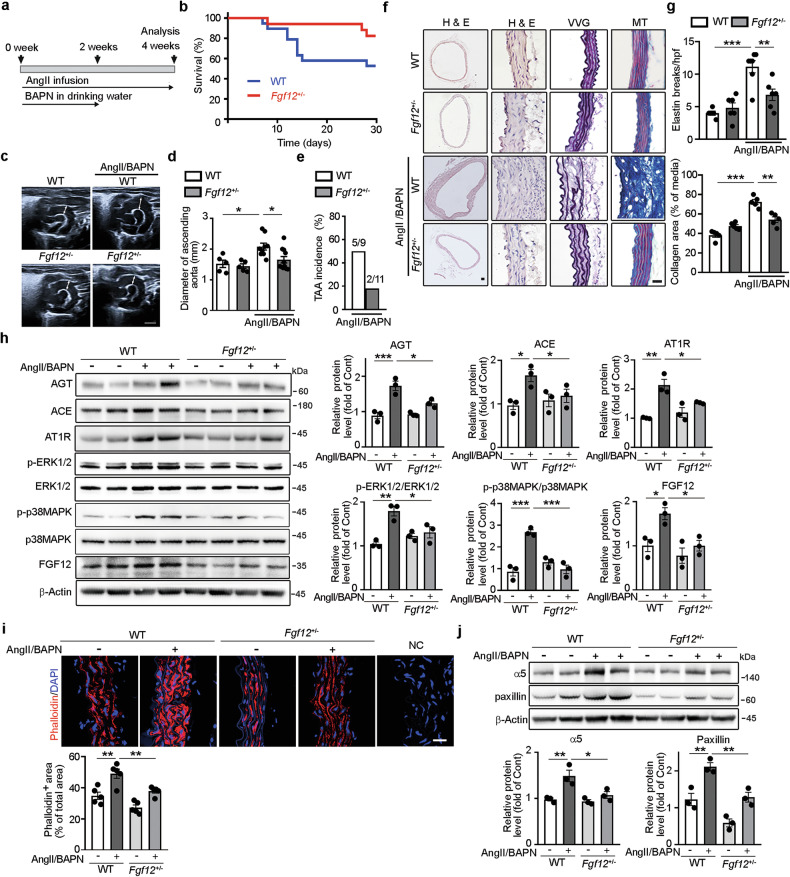
*Fgf12* haploinsufficiency ameliorates TAA formation and attenuates aberrant mechanosignaling in AngII/BAPN-challenged mice. **a** Experimental design: 12-week-old *Fgf12*^+/−^ and WT mice were challenged with AngII infusion and BAPN administration for 4 weeks. **b** Kaplan–Meier survival curves for WT and *Fgf12*^+/−^ mice following AngII/BAPN challenge (*n* = 17–19 mice per group). **c** Representative ultrasound images of the ascending aorta in WT and *Fgf12*^+/−^ mice with or without AngII/BAPN challenge. Scale bar, 2 mm. **d** Quantification of maximal diameters of ascending aortas (*n* = 5–11 mice per group). **e** Incidences of TAA in AngII/BAPN-challenged WT and *Fgf12*^+/−^ mice (*n* = 9–11 mice per group). **f** Representative H&E-, VVG- (elastin, black) and MT- (collagen, dark blue) stained images of ascending aortic sections from each group. Scale bars, 20 μm. **g** Quantification of elastin breaks per high-powered field (hpf) and collagen deposited area (% of total medial area) from images as in **f** (*n* = 5–7 mice per group). **h** Representative western blot images and quantification of AGT, ACE, AT1R, p-ERK1/2, p-p38MAPK and FGF12 in thoracic aortas. **i** Representative immunofluorescence images and quantification of phalloidin^+^ area (red) in ascending aortic sections (*n* = 3 mice per group). Nuclei (DAPI (blue); NC was stained with nonrelevant primary IgGs. Scale bar, 20 μm. **j** Representative western blot images and quantification of integrin α5 and paxillin expression in thoracic aortas. In **h** and **j**, phospho-protein levels were normalized to total protein. Protein levels are quantified relative to unchallenged WT mice (set as 1; *n* = 3 independent experiments). All data are presented as mean ± s.e.m. and were analyzed by one-way ANOVA with Bonferroni’s post-hoc test (*P < 0.05, **P < 0.01, ***P < 0.001).

To determine whether *FGF12*-driven AT1R signaling contributes to TAA development in vivo, we examined whether reduced FGF12 expression diminishes TAA incidence and severity in an established AngII-induced aneurysm model. *Fgf12*^+/−^ mice, which exhibit markedly reduced FGF12 expression but maintain normal BPs and cardiac function comparable to WT littermates, were used for these studies (Supplementary Figs. 7 and 8). WT and *Fgf12*^+/−^ mice were challenged with chronic AngII infusion in combination with BAPN administration for 4 weeks (Fig. 5a). This protocol reliably induces TAA and aortic rupture in mice^31–33^. Kaplan–Meier survival analysis revealed significantly lower mortality in AngII/BAPN-challenged *Fgf12*^+/−^ mice (17.6%, 3/17) compared with challenged WT mice (47.3%, 9/19), primarily due to a reduced incidence of aortic rupture (Fig. 5b). Ultrasound imaging demonstrated marked dilation of the ascending aorta in AngII/BAPN-challenged WT mice compared with unchallenged WT controls (Fig. 5c,d). By contrast, *Fgf12*^+/−^ mice showed significantly less aortic dilation following AngII/BAPN challenge. Consistently, the incidence of TAA was substantially lower in challenged *Fgf12*^+/−^ mice than in challenged WT mice (Fig. 5e). Histological analysis revealed that challenged WT mice displayed severe elastin fragmentation and extensive collagen deposition in the ascending aortas, whereas challenged *Fgf12*^+/−^ mice maintained a relatively preserved aortic wall architecture with minimal elastin breaks and collagen accumulation (Fig. 5f,g). We next examined whether *Fgf12* haploinsufficiency suppresses AngII/AT1R signaling and aberrant mechanosignaling in the thoracic aorta of AngII/BAPN-challenged mice. Challenged WT mice exhibited increased expression of AGT, ACE and AT1R, along with enhanced ERK1/2 and p38MAPK phosphorylation, compared with unchallenged WT controls (Fig. 5h). Notably, these changes were significantly attenuated in challenged *Fgf12*^+/−^ mice. Immunofluorescence staining showed increased phalloidin^+^ stress fibers in the ascending aortas of challenged WT mice, which was significantly diminished in challenged *Fgf12*^+/−^ mice (Fig. 5i). Likewise, western blot analysis demonstrated reduced integrin α5 and paxillin expression in the thoracic aortas of challenged *Fgf12*^+/−^ mice compared with challenged WT mice (Fig. 5j). Collectively, these findings demonstrate that *Fgf12* haploinsufficiency reduces TAA incidence and rupture, attenuates AT1R signaling and suppresses aberrant mechanosignaling in the thoracic aortas of AngII/BAPN-challenged mice.

### *Fgf12* haploinsufficiency mitigates TAA progression and alleviates dysregulated mechanosignaling in *Fbn1*^*C1039G/+*^ mice

**Fig. 6:**
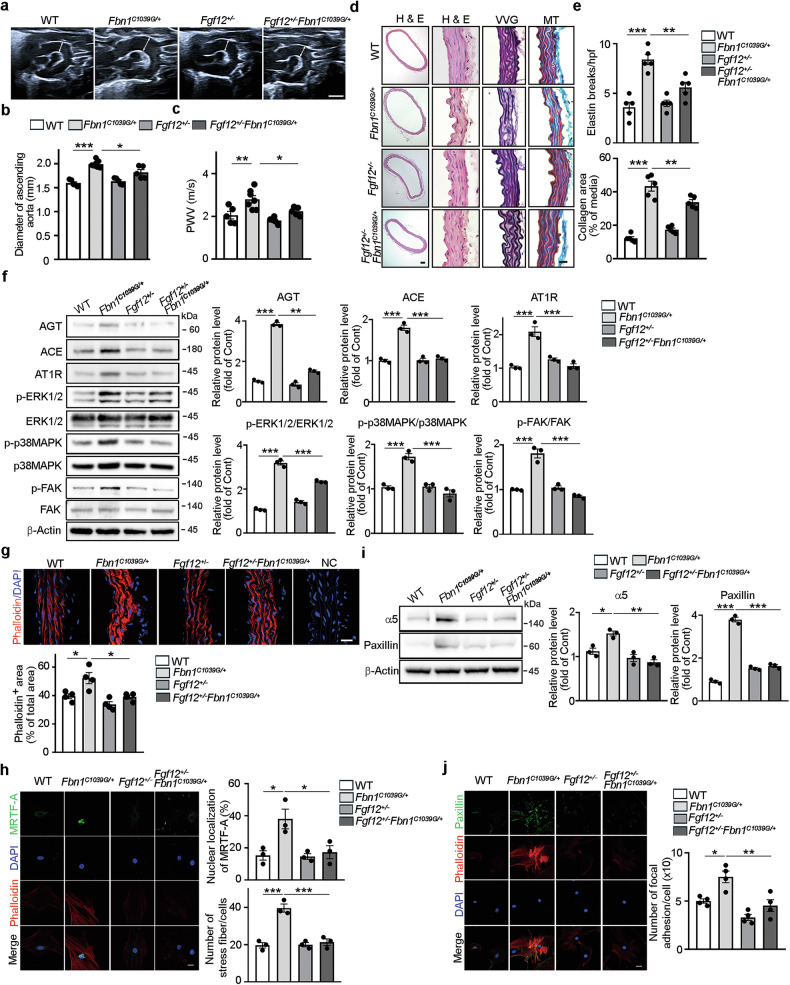
*Fgf12* haploinsufficiency mitigates TAA progression and alleviates dysregulated mechanosignaling in *Fbn1*^*C1039G/+*^ mice. **a**,**b** Representative ultrasound images and quantification of maximal diameters of ascending aortas in 16-week-old WT, *Fbn1*^*C1039G/+*^, *Fgf12*^+/−^ and *Fgf12*^+/−^*Fbn1*^*C1039G/+*^ mice (*n* = 5–8 mice per group). Scale bar, 2 mm. **c** PWV in the same four groups (*n* = 5–7 mice per group). **d** Representative H&E-, VVG- (elastin, black), and MT- (collagen, dark blue) stained images of ascending aortic sections from each group. Scale bars, 20 μm. **e** Quantification of elastin breaks per hpf and collagen deposited area (% of total medial area) from images as in **d** (*n* = 5 mice per group). **f** Representative western blot images and quantification of AGT, ACE, AT1R, p-ERK1/2, p-p38MAPK and p-FAK in thoracic aortas. **g** Representative immunofluorescence images and quantification of phalloidin^+^ area (red) in ascending aortic sections (*n* = 4 mice per group). Nuclei (DAPI, blue); NC was stained with nonrelevant primary IgGs. **h** Representative immunofluorescence images and quantification of MRTF-A nuclear translocation (green) and number of phalloidin^+^ stress fibers (red) in primary aortic SMCs isolated from ascending aortas of each group. **i** Representative western blot images and quantification of integrin α5 and paxillin expression in thoracic aortas. **j** Representative immunofluorescence images and quantification of number of paxillin^+^ FAs in aortic SMCs isolated from ascending aortas of each group. In **f** and **i**, phospho-protein levels were normalized to total protein. Protein levels are quantified relative to WT mice (set as 1; *n* = 3 independent experiments). In **g**, **h** and **j**, images and quantified data are representative of three to four independent experiments. Nuclei were stained with DAPI (blue). Scale bars, 20 μm. All data are presented as mean ± s.e.m. and were analyzed by one-way ANOVA with Bonferroni’s post-hoc test (*P < 0.05, **P < 0.01, ***P < 0.001).

We next generated *Fgf12*^+/−^*Fbn1*^*C1039G/+*^ mice to determine whether *Fgf12* haploinsufficiency mitigates TAA progression and alleviates aberrant mechanosignaling in *Fbn1*^*C1039G/+*^ mice. As previously reported, ultrasound imaging and PWV measurements showed that 12- and 16-week-old *Fbn1*^*C1039G/+*^ mice exhibited significant dilation of the ascending aortas and elevated arterial stiffness compared with age- and sex-matched WT mice^34,35^ (Fig. 6a–c and Supplementary Fig. 9). By contrast, *Fgf12*^+/−^*Fbn1*^*C1039G/+*^ mice displayed significantly smaller ascending aortic diameters and lower PWV values than sex-matched *Fbn1*^*C1039G/+*^ littermates. Histological analysis of ascending aortas revealed substantially reduced elastin degradation and collagen deposition in *Fgf12*^+/−^*Fbn1*^*C1039G/+*^ mice compared with *Fbn1*^*C1039G/+*^ mice (Fig. 6d,e). We then investigated whether *Fgf12* haploinsufficiency attenuates AngII/AT1R signaling and restores dysregulated mechanosignaling in the thoracic aorta of *Fbn1*^*C1039G/+*^ mice. Compared with WT controls, *Fbn1*^*C1039G/+*^ mice showed increased aortic expression of AGT, ACE and AT1R, as well as enhanced ERK1/2, p38MAPK and FAK phosphorylation (Fig. 6f). Notably, these changes were significantly attenuated in *Fgf12*^+/−^*Fbn1*^*C1039G/+*^ mice. Immunofluorescence staining of aortic tissue sections and of primary aortic SMCs isolated from ascending aortas revealed a significant increase in stress fiber density and nuclear translocation of MRTF-A in *Fbn1*^*C1039G/+*^ mice, whereas these pathological changes were substantially reduced in *Fgf12*^+/−^*Fbn1*^*C1039G/+*^ mice (Fig. 6g,h; see Supplementary Fig. 10 for enlarged images). Western blot analysis further showed reduced integrin α5 and paxillin expression in the thoracic aortas of *Fgf12*^+/−^*Fbn1*^*C1039G/+*^ mice compared with *Fbn1*^*C1039G/+*^ mice (Fig. 6i). Consistently, immunofluorescence staining of aortic SMCs isolated from ascending aortas confirmed a significant reduction in paxillin^+^ FA density in *Fgf12*^+/−^*Fbn1*^*C1039G/+*^ mice compared with *Fbn1*^*C1039G/+*^ mice (Fig. 6j; see Supplementary Fig. 11 for enlarged images). Collectively, these results suggest that *Fgf12* haploinsufficiency significantly attenuates progressive aortic dilation and arterial stiffening, while alleviating dysregulated mechanosignaling in the ascending aortas of *Fbn1*^*C1039G/+*^ mice.

## Discussion

Emerging evidence suggests that dysregulated mechanosignaling in aortic SMCs plays a critical role in the pathogenesis of inherited TAA, including MFS^36–38^. However, the molecular mediators driving this aberrant mechanosignaling have remained largely undefined. In the present study, we identified FGF12 as a novel regulator of pathological mechanosignaling in aortic SMCs and a key contributor to TAA development in MFS.

We found that FGF12 expression was markedly increased in the aortic SMCs of thoracic aneurysmal aorta from both *Fbn1*^*C1039G/+*^ mice and patients with MFS. Mechanistically, FGF12 expression was induced by TGF-β/SMAD signaling, which is activated in response to *Fbn1* mutations, as well as by cyclic mechanical stretch that imposes mechanical overload on the thoracic aorta in MFS. Adenoviral overexpression of FGF12 in HASMCs activated the AngII/AT1R/RhoA/ROCK signaling cascade. The RhoA/ROCK pathway is known to promote stress fiber formation, FA assembly and FAK signaling in vascular SMCs^39–41^. Consistent with these known effects, we observed that FGF12 overexpression enhanced F-actin formation, nuclear translocation of MRTF-A and phosphorylation of MLC and cofilin. It also increased the expression of FA constituents and activated FAK/SRC signaling in HASMCs. Moreover, we found that treatment with a FAK inhibitor significantly reduced the level of GTP-bound, active RhoA in Ad *FGF12*-transduced HASMCs (Supplementary Fig. 12). This observation is consistent with reports that FAK regulates the activities of GTPase-activating proteins and guanine nucleotide exchange factors, and it suggests the existence of a positive feedback loop between integrin-mediated FAK and RhoA/ROCK pathways in HASMCs^42^. In vivo experiments using *Fgf12*^*+/−*^ mice demonstrated that *Fgf12* haploinsufficiency significantly attenuated AT1R signaling and reduced aberrant stress fiber and FA formation in the ascending aortas of both AngII/BAPN-challenged and *Fbn1*^*C1039G/+*^ mice. These findings align with previous reports showing elevated RhoA-GTP levels, increased actin polymerization and upregulated integrin expression in vascular SMCs from patients with MFS, further supporting a pathological role for FGF12 in MFS-associated TAA^6–8^.

We also found that FGF12 increased the stiffness of aortic SMCs in an AT1R/ROCK-dependent manner. Cellular stiffness is regulated by both FAs and the actin cytoskeleton. FA formation and FAK activation enhance cell–ECM adhesion, thereby limiting cellular deformability in response to external mechanical forces. Notably, α5β1 integrin, a major fibronectin-binding integrin, may play a key role in regulating the adhesive properties of aortic SMCs in aneurysmal aortas, where fibronectin expression is elevated^43,44^. In parallel, actin cytoskeletal remodeling, particularly G-actin to F-actin polymerization, is another major determinant of cellular stiffness, contributing to the generation of intracellular mechanical tension^45^. A recent study further demonstrated that increased cytoskeleton tension via upregulation of α-actinin 2, an F-actin crosslinking protein, promotes vascular SMC stiffness in an AngII-induced murine model of aortic aneurysm^9^. Importantly, vascular SMCs with elevated stiffness exhibit impaired mechanosensation and a reduced ability to generate contractile force in response to mechanical stress. Such dysfunctional mechanosensation may lead to misinterpretation of elevated wall stress as low stress, ultimately resulting in maladaptive weakening of the aortic wall^46^. In this regard, FGF12-mediated increases in FA assembly, FAK signaling and stress fiber formation may promote pathological stiffening and defective mechanosensation in aortic SMCs, thereby contributing to progressive aortic dilation.

Our data show that *Fgf12* haploinsufficiency significantly mitigated TAA development and progression in both AngII/BAPN-challenged and *Fbn1*^*C1039G/+*^ mice, accompanied by reduced elastin fragmentation and collagen deposition in the thoracic aortas. These results highlight FGF12 as a potential therapeutic target for preventing or stabilizing TAA growth. In addition, *Fgf12*^+/−^*Fbn1*^*C1039G/+*^ mice exhibited reduced PWV, an in vivo index of arterial stiffness, compared with age- and sex-matched *Fbn1*^*C1039G/+*^ mice. Large arterial stiffness reflects the reduced distensibility of the aortic wall in response to applied pressure. Increased stiffness of the proximal aorta accelerates pressure wave propagation, thereby imposing elevated cyclic mechanical load on the thoracic aorta. Indeed, recent clinical studies have identified aortic stiffness as an early independent predictor of aortic dilation rate and dissection risk in patients with MFS^47–49^. Traditionally, arterial stiffening has been attributed to ECM remodeling, particularly elastin degradation and collagen accumulation. However, emerging evidence increasingly recognizes vascular SMC stiffness as an additional determinant of arterial stiffness^38,50,51^. For example, Qiu et al. demonstrated that both ECM alterations and increased vascular SMC stiffness contribute to thoracic aortic stiffening in aging nonhuman primates^20^. Similarly, studies in hypertensive mouse models revealed that intrinsic vascular SMC stiffness promotes large-artery stiffness independently of ECM changes^52,53^. In light of these findings, our data suggest that FGF12 may promote arterial stiffening in *Fbn1*^*C1039G/+*^ MFS mice, at least in part by enhancing the intrinsic stiffness of aortic SMCs. Nonetheless, since *Fgf12* haploinsufficiency also reduced elastin fragmentations and collagen deposition, further studies are needed to delineate the relative contribution of intrinsic cellular stiffness- versus ECM-driven mechanisms in FGF12-mediated arterial stiffening.

Taken together, our study demonstrates that FGF12 is a key mediator of aberrant mechanosignaling in aortic SMCs during TAA development in MFS. It also suggests that FGF12 knockdown may offer a promising therapeutic strategy to prevent or slow TAA progression in MFS. Given the role of aberrant mechanosignaling and vascular SMC stiffness in various vascular diseases, further investigations are warranted to determine whether FGF12 also contributes to the pathogenesis of hypertension and age-related arterial stiffening.

## Supplementary information

Supplementary Information

## Data Availability

The data underlying this article will be shared on reasonable request to the corresponding author.
