# Supplementary material for: FGF12 induces aberrant mechanosignaling in aortic smooth muscle cells during thoracic aortic aneurysm formation in Marfan syndrome mice
Source: Exp Mol Med. 2026 Jan 16;58(1):199–210. doi: 10.1038/s12276-025-01621-y (PMC12868687; doi:10.1038/s12276-025-01621-y)
Supplement: Supplementary file 1 — Supplementary Information [file 12276_2025_1621_MOESM1_ESM.pdf]

Supplementary Information

FGF12 Induces Aberrant Mechanosignaling in Aortic Smooth Muscle Cells During Thoracic Aortic Aneurysm Formation in Marfan Syndrome Mice

Koung Li Kim[1], Minju Kim[1], Yubin Hwang[1], Duk-Kyung Kim[2], Jeongmin Kim[1], June Hyeok Lee[1], Yong-Wook Son[3], Jae-Hyung Jang[3,6], Kyung-Sun Heo[4], Misato Iwashita[5], Yoichi Kosodo[5], Wonhee Suh[1]*

[1]College of Pharmacy, Chung-Ang University, Seoul 06974, Korea
[2]Division of Cardiology, Department of Medicine, Samsung Changwon Hospital, Sungkyunkwan University School of Medicine. Gyeongsangnam-do 51353, Korea
[3]Department of Chemical and Biomolecular Engineering, Yonsei University, Seoul 03722, Korea.
[4]College of Pharmacy, Chungnam National University, Daejeon 34134, Korea
[5]Neural Regeneration Lab, Korea Brain Research Institute, Daegu 41062, Korea
[6]R&D Center, GluGene Therapeutics Inc., Seoul 03722, Korea

[*]Address for Correspondence:
Wonhee Suh, PhD
College of Pharmacy, Chung-Ang University,
84 Heukseok-ro, Dongjak-gu, Seoul 06974, Korea.
Tel: 82-2-820-5960; Fax: 82-2-816-7338; E-mail: wsuh@cau.ac.kr

Supplementary Figures and Figure Legends

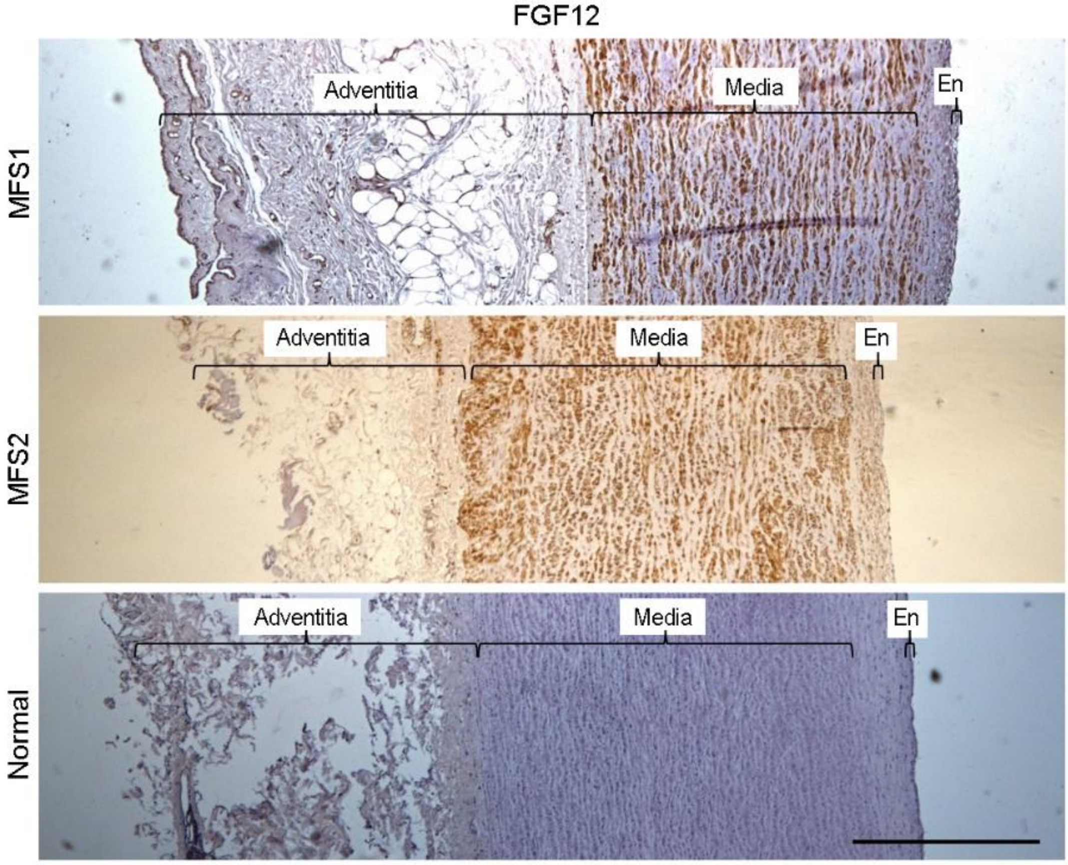

Supplementary Fig. 1 Expression of FGF12 in medial SMCs of thoracic aneurysmal aortas from patients with MFS.

Representative immunohistochemical images (FGF12, brown) of thoracic aortic tissues from a normal subject and two patients with MFS (MFS1, MFS2). En denotes endothelium. Scale bar = 500 μm.

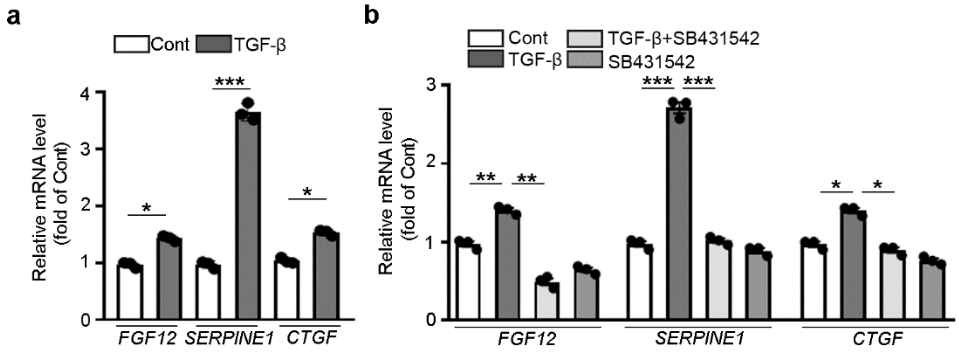

Supplementary Fig. 2 TGF-β/SMAD signaling increases *FGF12* mRNA levels in human aortic smooth muscle cells.

a, **b** Human aortic smooth muscle cells were treated with or without TGF-β (10 ng/mL) and ALK5 inhibitor (SB431542, 10μM) in smooth muscle growth medium for 48 h. mRNA levels of *FGF12* and TGF-β target genes (*SERPINE1*, *CTGF*) were assessed using real-time RT-PCR analysis. Data are expressed relative to untreated controls (Cont, set as 1; $n = 3$ independent experiments). All data are presented as mean ± SEM. Statistical analysis was determined using one-way ANOVA with Bonferroni's *post hoc* test (*$p < 0.05$, **$p < 0.01$, ***$p < 0.001$).

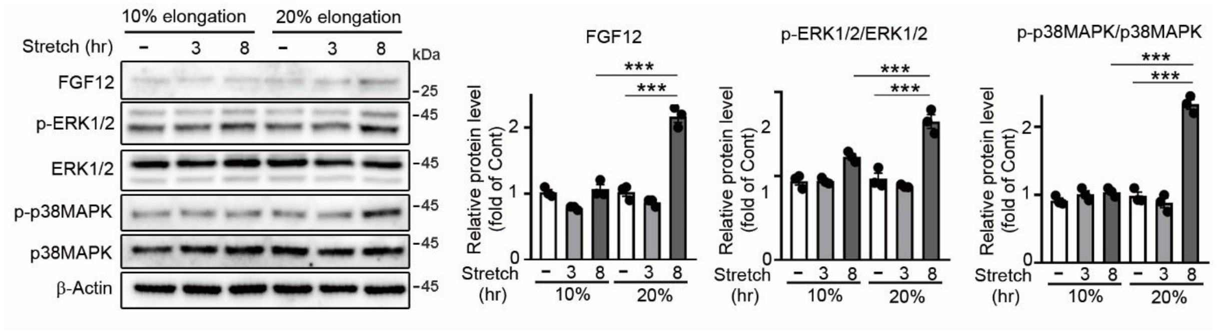

Supplementary Fig. 3 FGF12 expression in rat aortic SMCs subjected to physiological and pathological levels of cyclic mechanical stretch.

Rat aortic SMCs were subjected to cyclic mechanical stretch at 10% (physiological) or 20% (pathological) elongation (1.0 Hz) for 0, 3, or 8 h using the FlexCell system. Western blotting was performed to evaluate protein levels of FGF12, phosphorylated ERK1/2 (p-ERK1/2), total ERK, phosphorylated p38MAPK (p-p38MAPK) and total p38MPAK. Phospho-protein levels were normalized to total protein levels. Protein levels are quantified relative to unstretched controls (Cont, set as 1; *n* = 3 independent experiments). All data are presented as mean ± SEM and were analyzed by one-way ANOVA with Bonferroni's *post hoc* test (***$p <$ 0.001).

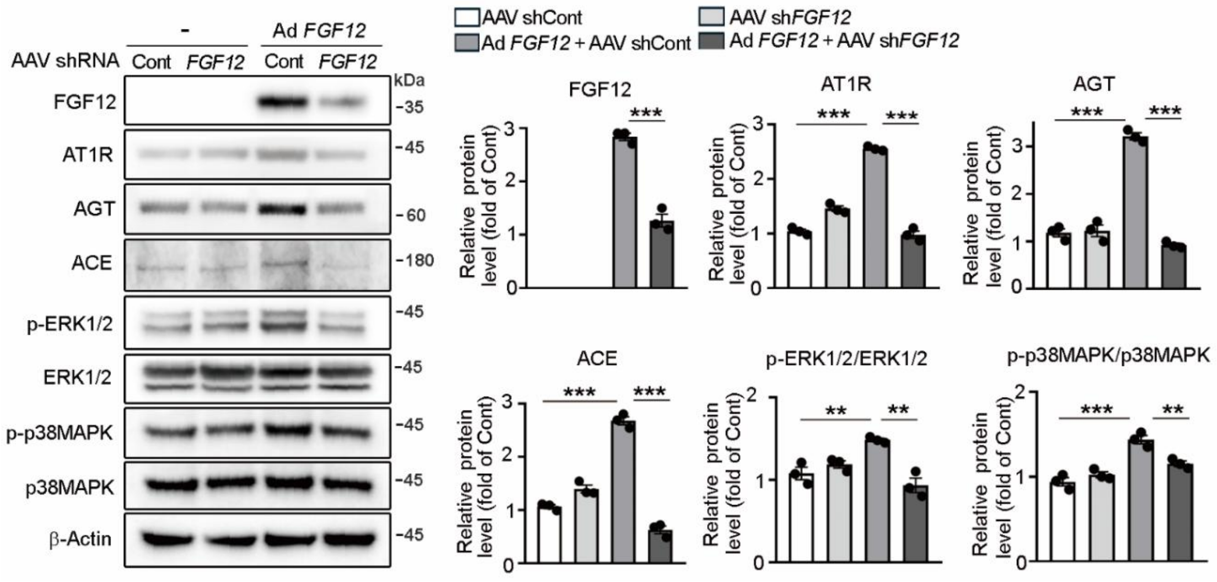

Supplementary Fig. 4 shRNA-mediated knockdown of *FGF12* reverses *FGF12* overexpression-induced changes in the AT1R/AngII pathway in HASMCs.
HASMCs were transduced with adenovirus expressing FGF12 (Ad *FGF12*); subsequently, cells were treated with either an AAV expressing a control shRNA (shCont) or an AAV expressing an shRNA targeting *FGF12* (AAV sh*FGF12*). Phospho-protein levels were normalized to total protein levels. Protein levels are expressed relative to untransduced controls (Cont, set as 1; $n = 3$ independent experiments). All data are presented as mean ± SEM and were analyzed by one-way ANOVA with Bonferroni's *post hoc* test (**$p < 0.01$, ***$p < 0.001$).

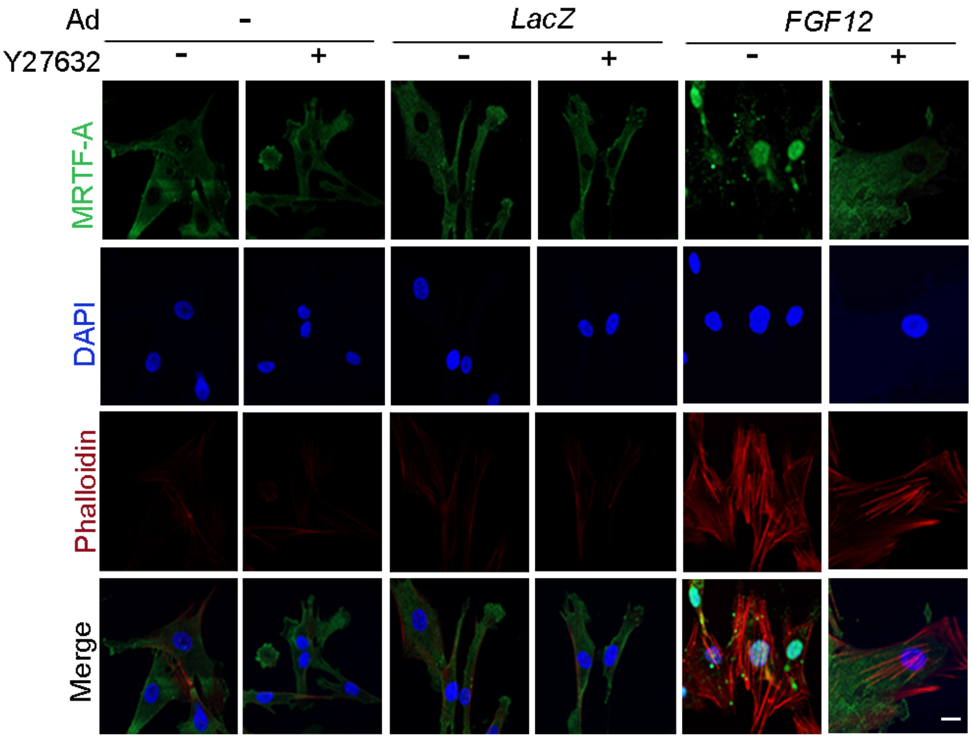

Supplementary Fig. 5 Enlarged immunnofluorescent images in Fig. 4d.

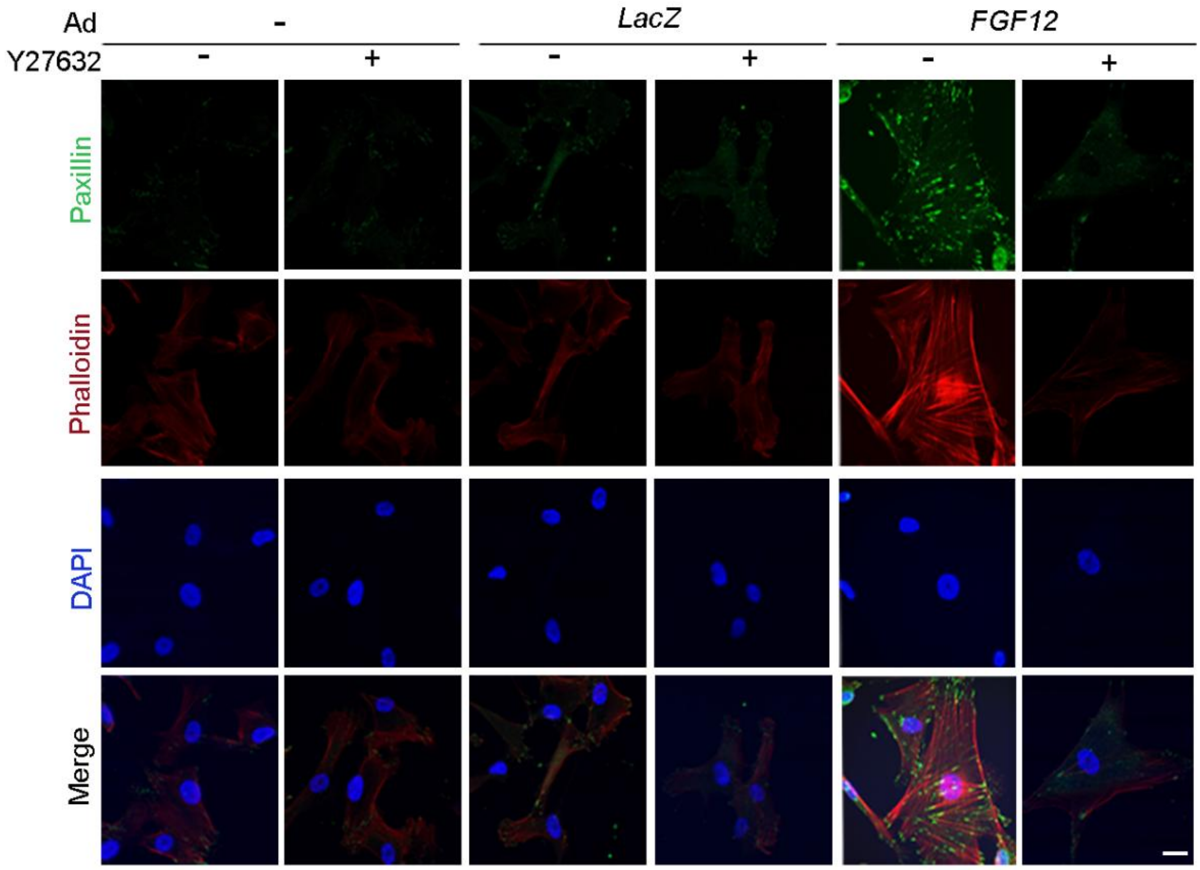

Supplementary Fig. 6 Enlarged immunnofluorescent images in Fig. 4f.

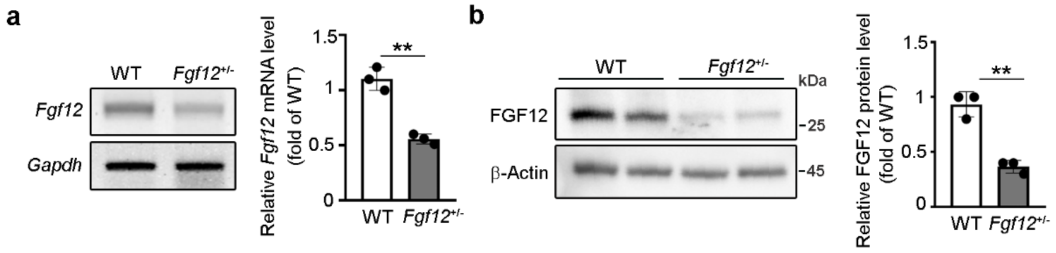

Supplementary Fig. 7 Expression of FGF12 in the thoracic aorta of wild type and *Fgf12*$^{+/-}$ mice.

mRNA (**a**) and protein (**b**) levels of *Fgf12* in the thoracic aorta of wild type (WT) and *Fgf12*$^{+/-}$ mice were measured using RT-PCR (**a**) and western blotting (**b**). Data are expressed relative to WT mice (set as 1). *Gapdh* and β-actin were used as loading controls. All data are presented as mean ± SEM and were analyzed by one-way ANOVA with Bonferroni's *post hoc* test (**$p < 0.01$; $n = 3$ independent experiments).

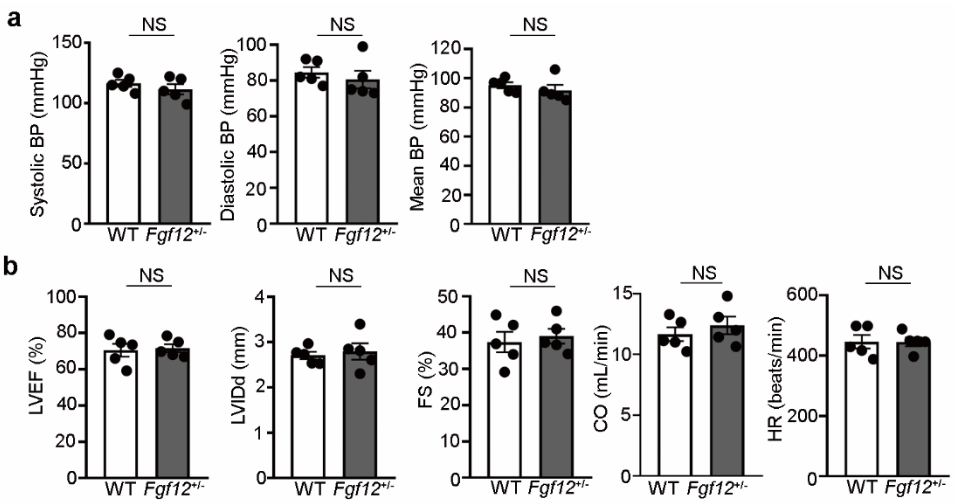

Supplementary Fig. 8 Blood pressure and cardiac function are comparable between wild-type and *Fgf12*$^{+/-}$ mice.

a Systolic, diastolic, and mean arterial blood pressure (BP) in wild-type (WT) and *Fgf12*$^{+/-}$ mice measured by tail plethysmography. **b** Cardiac function parameters including left ventricular ejection fraction (LVEF), left ventricular internal diameters in diastole (LVIDd), fractional shortening (FS), cardiac output (CO), and heart rate (HR) in WT and *Fgf12*$^{+/-}$ mice measured by echocardiography. Data are presented as mean ± SEM (n = 5 mice per group); no significant (NS) differences were found between groups (unpaired student's t-test).

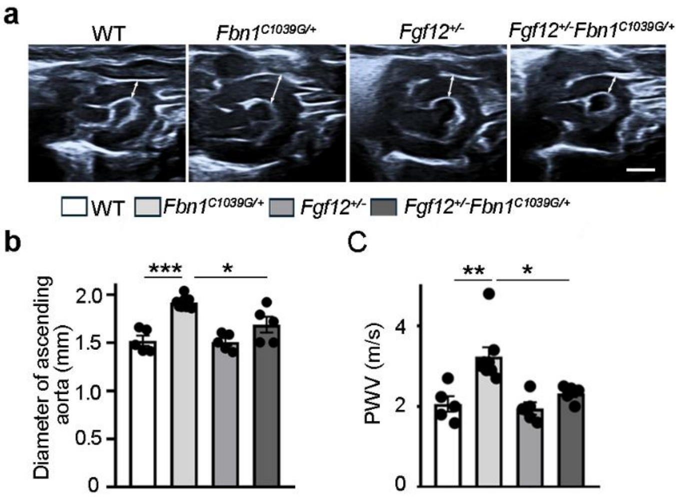

Supplementary Fig. 9 Quantification of maximal ascending aortic diameters and arterial stiffness in WT, *Fbn1$^{C1039G/+}$*, *Fgf12$^{+/-}$*, and *Fgf12$^{+/-}$Fbn1$^{C1039G/+}$* mice at 12 weeks of age. **a**, **b** Representative ultrasound images and quantification of maximal diameters of ascending aortas in 12-week-old wild type (WT), *Fbn1$^{C1039G/+}$*, *Fgf12$^{+/-}$*, and *Fgf12$^{+/-}$Fbn1$^{C1039G/+}$* mice. Scale bar = 2 mm. **c** Pulse wave velocity (PWV) in the same four groups. All data are presented as mean ± SEM and were analyzed by one-way ANOVA with Bonferroni's *post hoc* test (*$p < 0.05$, **$p < 0.01$, ***$p < 0.001$; n = 5–8 mice per group).

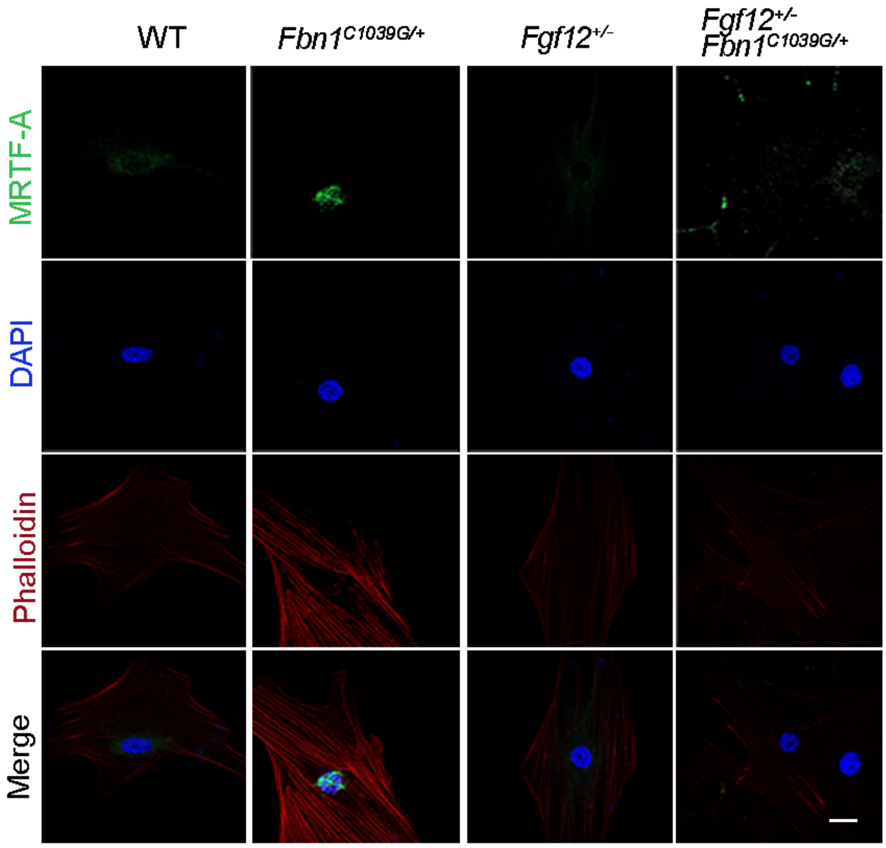

Supplementary Fig. 10 Enlarged immunnofluorescent images in Fig. 6h.

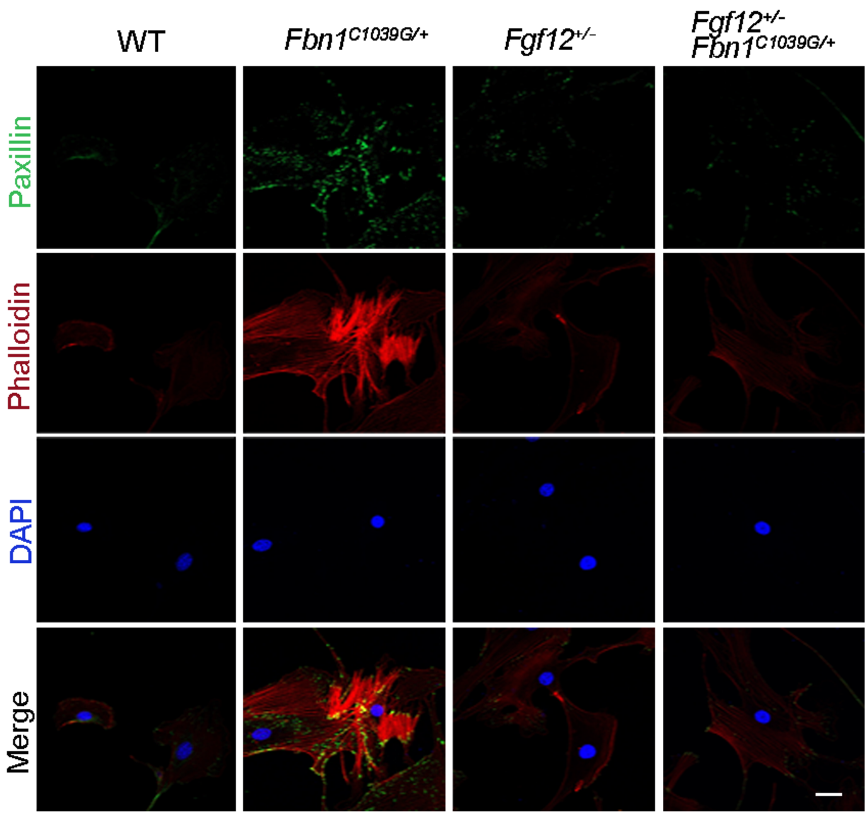

Supplementary Fig. 11 Enlarged immunnofluorescent images in Fig. 6j.

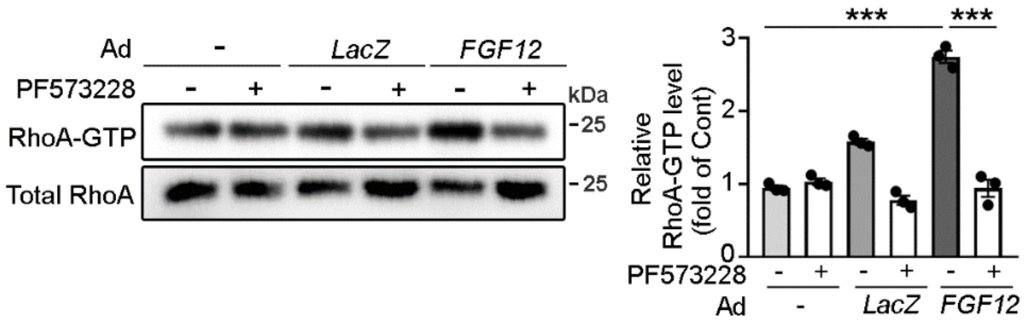

Supplementary Fig. 12 FAK inhibition reduces *FGF12*-induced RhoA activation in HASMCs.

Following adenoviral transduction (Ad *LacZ*, Ad *FGF12*), HASMCs were treated with or without a FAK inhibitor (PF573228, 10 μM). GTP-bound RhoA (RhoA-GTP) levels were quantified by RhoA pull-down assays, normalized to total RhoA, and are expressed relative to untransduced controls (Cont, set as 1; $n = 3$ independent experiments). All data are presented as mean ± SEM and were analyzed by one-way ANOVA with Bonferroni's *post hoc* test (***$p < 0.001$).

Supplementary Table 1 List of primer sequences.

Target gene	Forward sequence (5' to 3')	Reverse sequence (5' to 3')
FGF12(human), *Fgf12*(mouse)	GCGATAGCCAGCTCCTTGAT	GAAGCGCACTTTGCTGAACA
SERPINE1	CCTGGTTCTGCCCAAGTTCT	ATGCGGGCTGAGACTATGAC
CTGF	TTAGCGTGCTCACTGACCTG	GCCACAAGCTGTCCAGTCTA
GAPDH (human)	AACCTGCCAAATATGATGAC	TCATACCAGGAAATGAGCTT
Gapdh (mouse)	ATGACTCCACTCACGGCAAA	ATGATGACCCTTTTGGCTCC

Supplementary Table 2 List of IgGs used in western blot and immunostaining analyses.

Target antigen	Dilution	Vendor or Source	Catalog #
PAI-1	1:1000	Thermo Scientific (Rockford, IL, USA)	MA5-17171
CTGF	1:1000	R&D systems (Minneapolis, MN, USA)	4376
α-smooth muscle actin	1:1000	Sigma-Aldrich (St. Louis, MO, USA)	C6198
p-SMAD2	1:1000	Cell signaling (Danvers, MA, USA)	3108
FGF12	1:1000	Abcam (Cambridge, UK)	ab231956
p-ERK1/2	1:1000	Cell signaling	4370
ERK1/2	1:1000	Cell signaling	9102
p-p38MAPK	1:1000	Cell signaling	4511
p38MAPK	1:1000	Cell signaling	9212
β-Actin	1:2000	Santa Cruz Biotechnology (Dallas, TX)	sc47778
MRTF-A	1:100	Santa Cruz Biotechnology	SC-21558
AGT	1:1000	R&D systems	AF3156
ACE	1:1000	Abcam	ab254222
AT1R	1:1000	Abcam	ab124505
p-MLC	1:1000	Cell signaling	3671
MLC	1:1000	Cell signaling	8905
p-Cofilin	1:1000	Abcam	ab283500
Cofilin	1:1000	Abcam	ab42824
p-SRC	1:1000	Cell signaling	2101
SRC	1:1000	Cell signaling	2109
p-FAK	1:1000	Abcam	ab24781
FAK	1:1000	Cell signaling	3285
α5 integrin	1:1000	Abcam	ab150361
β1 integrin	1:1000	Abcam	ab30394
paxillin	1:1000	Abcam	ab32084

Goat anti-rabbit antibody, Peroxidase	1:400	Vector Laboratories (Burlingame, CA, USA)	PI-1000
Goat anti-mouse antibody, Peroxidase	1:400	Abcam	ab023
Alexa Fluor® 488 goat anti-rabbit antibody	1:400	Thermo Scientific	A11008
Alexa Fluor® 488 goat anti-mouse antibody	1:400	Thermo Scientific	A11001
Alexa Fluor® 594 rabbit anti-goat antibody	1:400	Thermo Scientific	A11080
